# Bee Sting Venom as a Viable Therapy for Breast Cancer: A Review Article

**DOI:** 10.7759/cureus.54855

**Published:** 2024-02-25

**Authors:** Aabhas Bindlish, Anupama Sawal

**Affiliations:** 1 Anatomy, Jawaharlal Nehru Medical College, Datta Meghe Institute of Higher Education and Research, Wardha, IND

**Keywords:** cancer genomics, chemotherapy, apitherapy, bee venom, breast cancer

## Abstract

Breast cancer is a kind of aggressive cancer that significantly affects women worldwide, thus making research on alternative and new therapies necessary. The potential impact of bee venom on breast cancer is the main subject of this analysis of this research article. Bee venom has drawn the attention of the world with the help of its constituent ingredients, namely the bioactive compounds, enzymes, and complex blend of proteins. They have a particularly varied chemical makeup and proven anti-cancer capabilities. This is a detailed review demonstrating the components of bee venom and their individual functions in fighting cancer, as well as the results of previously conducted in-vitro and in-vivo research. As described later, bee venom has given positive results in triggering apoptosis, preventing cell migration, inhibiting metastasis and invasion, and suppressing the existing breast cancer cells. It is found to have worked better along with the already existing chemotherapy treatments. These results were also proved with the help of various animal studies that showed reduced tumor development, reduced metastasis, and improved therapeutic effectiveness. Furthermore, certain studies and case reports from all over the world have exhibited consistent results in females affected by breast cancer. This study found that people receiving chemotherapy experienced improved health outcomes and reduced discomfort, with fewer negative side effects. It is important to conduct extensive research on the safety and effectiveness of this treatment because it is yet to be approved. The ideal dosage and administration methods must be explored in clinical trials. Moreover, it is imperative to evaluate the results of any combined treatments with current medications. There should be constant monitoring to prevent any potential side effects. Other important things like allergic reactions and hidden concerns should also be considered.

## Introduction and background

The western honeybee, Apis mellifera, creates venom, a complex compound that aids in the treatment of a number of ailments, including pain [1], inflammation [2], and cancer [3]. Bee venom contains a variety of peptides, including melittin and apamin, as well as enzymes, including phospholipase A2 and hyaluronidase, physiologically active amines, including histamine and epinephrine, and non-peptides, including amino acids [4]. Melittin is one of them and makes up 40-50% of the dry weight of bee venom, making it a significant molecule. Other significant ingredients in dried bee venom include phospholipase A2, which makes up 10-12%, and apamin, which makes up 2-3% [5]. In this review article, we try to elaborate on the therapeutic effects of bee sting venom in suppressing tumor cells and lean more towards the treatment of breast cancer.

## Review

Breast cancer

Breast cancer is a disorder wherein the cells in the breast proliferate uncontrollably. There are various varieties and types of breast cancer. The type of breast cancer is determined by the rate at which breast cells turn malignant. The breast consists of three basic components, namely the connective tissue, the ducts, and the lobules. The lobules have the important function of producing milk that nourishes the newborn baby. The milk traverses through the ducts to the nipples, from where it is sucked out. The connective tissue, which is a layer of fat and fibrous tissue, acts as an envelope by holding everything in place. Breast cancer initiation takes place in these milk-traversing tubes, called ducts. For breast cancer to metastasize outside the body, it uses the pathways of lymph nodes and blood [6].

Breast cancer can affect both men and women, but it is considerably more common in women. In the US, male breast cancer affects roughly 2,600 men each year, making up less than 1% of all cases. Breast cancer is more common in transgender women than in cisgender men. In comparison to cisgender women, transgender men had a lower risk of developing breast cancer. Although it can happen at any age, breast cancer is most frequently discovered in adults over 50 years of age [7].

The most frequent reason why women seek medical attention for a breast issue is the discovery of a mass. Another frequently presented issue is breast soreness and pain. Discharge through nipples is also a condition that is seen commonly. Breast cancer discharges are highly spontaneous and red; they are occasionally accompanied by a tumor and are restricted to a single duct of a single breast. Malignancies of breast cancer usually show erythema and edema, as well as retraction of the skin or nipple. A lump suspected of breast cancer is commonly lone, distinct, and hard. Whereas in other cases, the lump is anchored to the muscle underneath or the skin.

**Figure 1:**
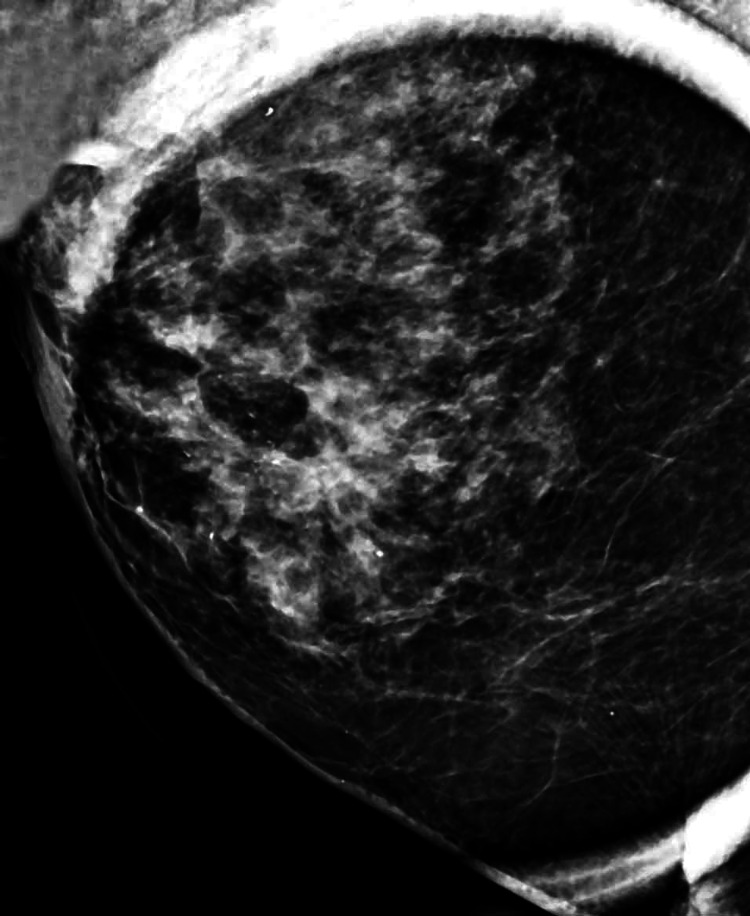
Mediolateral oblique view of the right breast mammograph Image is obtained by the author, Aabhas bindlish with the consent of the patient.

A suspicious lump is typically non-tender and unilateral. Sometimes, a thickening that does not correspond to a distinct tumor could be cancerous. When breast cancer is first discovered, it is rarely bilateral. Whether the woman is standing or lying down, the breasts should be inspected with her hands behind her head. Examining the breasts should include looking for changes in size, nipple or skin retractions, noticeable vein patterns, and indications of inflammation. Use your fingertips' flat surfaces to feel the breast tissue against the chest wall. Examine the axillary and supraclavicular regions for adenopathy. To check for discharge, just gently grip and massage the nipple [8]. Figure 1 below shows the mediolateral oblique view of the right breast lump of a 71-year-old woman.

Standard treatment option

Surgery, chemotherapy, endocrine therapy, radiation therapy, or a combination of these are the tried-and-true treatment modalities that have been in use for many years [9].

Taking medications that either lower the levels of circulating estrogen by blocking the conversion of androgens to estrogen or that impede estrogen's competitive binding to its receptors are both components of standard endocrine therapy. Some unfavorable effects or side effects of these medications include osteoporosis, hot flashes, arthralgia, myalgia, and uterine cancer. Chemotherapy is a crucial component of treatment for preventing recurrence because it prevents DNA replication, or mitosis. Asthenia, edema, myalgia, and leukemia are common complaints among patients receiving this treatment [10].

The spread of breast cancer, whether it’s limited to a local area or if it has infected the entire breast with the axillary lymph node, dictates the surgical treatment that needs to be incorporated to treat the patient [11]. By interfering with the lymphatic drainage system or injuring nerves, surgery can cause lymphedema [12].

The absolute survival benefit is increased, and the chance of local recurrence is decreased with radiation therapy, especially post-mastectomy radiation therapy [13]. However, a 10-year study revealed loco-regional recurrence and confirmed arm lymphedema complaints, including severe symptoms [14]. Cancer patients look to complementary and alternative medicine to lessen the negative effects of these conventional medicines.

The main compound of bee venom

Bees usually use their venom to protect themselves from predators. It is a transparent, acidic liquid with no smell that is made out of a hydrolytic mixture of proteins. Its pH ranges from 4.5 to 5.5.

Only 0.1 g of dry venom makes up one drop of bee venom, which is made up of 88% water. The latter peptide mixture contains peptides like melittin, adolapin, apamin, and mast cell degranulating peptides and is exceedingly complicated [15]. Table 1 describes the major peptide compounds of bee venom and their respective effects.

**Table 1:** Main compounds of bee venom

Peptide Compounds in Bee Venom	Biological Effects
Melittin	Melittin induces a range of cell death processes, including apoptosis, suppression of cancer motility, migration, metastasis, invasion, and inhibition of angiogenesis or proliferation. It also causes a cell cycle arrest [16].
Apamin	Through the mitochondria-related apoptotic pathway, apamin decreases apoptosis. Furthermore, apamin therapy significantly reduced apoptotic cell death in atherosclerosis-prone mouse models [17].
Adolapin	Adolapin is a peptide derived from bee venom that inhibits the activity of phospholipase A2 and has anti-inflammatory, antipyretic, and analgesic properties. The cyclooxygenase inhibitory qualities of this chemical cause it to have the desired effects by inhibiting the production of prostaglandins [18].
MCD Peptide	By releasing histamine, mast cell degranulation (MCD) peptides can promote mast cell degranulation, and at greater concentrations, they exhibit anti-inflammatory properties. K+ channels that depend on voltage are both bound and deactivated [19].
Protease inhibitor	Protease inhibitors have anti-rheumatic and anti-inflammatory effects. It blocks the actions of plasmin, thrombin, chymotrypsin, and trypsin [20].

Role of mellitin

Melittin, a cytolytic peptide generated from bee venom that has undergone extensive research, is regarded as a model for both cationic and other cytolytic peptides. It's intriguing how effective it is against viruses, bacteria, fungi, parasites, and tumors over a broad spectrum of conditions [21]. Apoptosis, reduction of angiogenesis or proliferation, cell cycle arrest, inhibition of cancer motility, migration, metastasis, and invasion are only a few of the several cell death processes that melittin produces [22].

Cell lysis is brought about by phospholipid bilayer breakdown, pore formation, and permeability induction during apoptosis. It has been demonstrated that melittin inhibits the proliferation of AGS cells by inducing time- and dose-dependent necrosis and apoptosis in gastric cancer cells. Cell shrinkage, cell shape irregularity, cellular separation, and membrane damage were used to illustrate these effects [23]. In SGC-7901 gastric cancer cells, melittin also triggered apoptosis through mitochondrial mechanisms [24].

Despite the fact that melittin is reputed to kill cancer cells by causing apoptosis, investigations into visualization have been fairly few. The addition of melittin to lipid monolayers was observed to cause distinct morphological alterations and apparent pore formation in recent atomic force microscopy (AFM) investigations [25].

Role of apamin

An 18-amino acid peptide called apamin has two disulfide bridges in it. Bee venom is the smallest neurotoxic [4]. Because this polypeptide can pass the blood-brain barrier, it can have a variety of effects on how the central nervous system functions. For instance, it has neurotoxic effects on the mammalian spinal cord, causing convulsions and hyperactivity. The potassium ion (K+) permeability of the cell membrane can also be influenced by apamin via inhibiting calcium-activated K+ channels. The toxin has the ability to prevent vascular smooth muscle cell migration and proliferation in the vascular smooth muscle [26].

The potential of apamin in atherosclerosis treatment approaches is highlighted by this finding. According to another study that examined the effects of K+ channel sensitivity to apamin, the neurotoxin can prevent non-pregnant women's myometrium from relaxing on its own due to nitric oxide [27].

Role of adolapin

With 103 amino acid residues, adolapin is a basic polypeptide. It is equivalent to 1% of bee venom's dry weight. According to research, adolapin inhibits cyclooxygenase activity and prostaglandin synthesis, which results in antipyretic, anti-inflammatory, and anti-nociceptive effects. The polypeptide may have analgesic properties and can inhibit the human platelet lipoxygenase enzyme [28]. The polypeptide may exert an analgesic effect by inhibiting lipoxygenase in human platelets.

Role of mast cell degranulating peptide

It is a polypeptide from bee venom that has 22 amino acids and a structure comparable to apamin since they both include two disulfide links. It makes between 2% and 3% of the dry weight of BV. The biological process by which mast cells release histamine is reflected in the name of MCD. It is an important K+ channel inhibitor, an epileptogenic neurotoxic, and can significantly lower rats' blood pressure [29]. MCD is a potent anti-inflammatory agent that may be a possibility for research into the secretory processes of inflammatory cells such as leukocytes, mast cells, and basophils in order to develop drugs with therapeutic uses [30].

Studies conducted on bee venom

The effect of bee venom on breast cancer cells has been studied in a number of in vitro experiments. According to these studies, bee venom can stop breast cancer cells from growing, cause apoptosis, and hinder cell invasion and migration. Bee venom has also been proven to work in concert with chemotherapeutic medications to increase their cytotoxicity and decrease drug resistance. Studies on animals have delved deeper into how bee venom affects breast cancer. These studies have shown that bee venom can decrease metastasis, inhibit tumor growth, and increase the effectiveness of conventional therapies. Bee venom delivery has also demonstrated a low level of toxicity to healthy tissues, underscoring its potential as an adjuvant treatment [3].

Apitherapy 

Apitherapy is a type of alternate medicine in which honeybee products like pollen, honey, jelly, propolis, and primarily bee venom, also called Apitoxin [31].

Even though cancer research has advanced dramatically, traditional therapy falls short of fully managing cancer. Bees convert plant sap and gums into the resinous material known as propolis, often known as bee glue. Despite being used as a medicinal agent since ancient times, it hasn't gained widespread acceptance as a health promoter. Its pharmacological properties have been confirmed, and they include anti-inflammatory, antidiabetic, dermato-protective, anti-allergic, laxative, immunomodulatory, anticancer, and antibacterial. Cancers of the skin, breast, liver, pancreas, kidney, bladder, prostate, colon, brain, head and neck, and blood have been successfully treated with propolis. The primary mechanisms of controlling cancer have been identified as matrix metalloproteinase inhibition, anti-angiogenesis, metastasis prevention, cell-cycle arrest, activation of apoptosis, and moderation of chemotherapy-induced adverse side effects [32].

Apitherapy is a young field that has the potential to have a global impact on the financial elements of cancer research, especially in underserved areas. Melittin and honeybee venom's molecular mechanisms of action have not yet been fully analyzed in studies, and as a result, their best uses in the field of oncology have not yet been thoroughly examined. This is especially true for the treatment of breast cancer, which is the most common cancer in women worldwide [33].

Clinical trials and human studies

Bee venom effectiveness in breast cancer patients has been studied in a small number of clinical trials and case reports. Positive results from this research include increased quality of life, less pain, and fewer chemotherapy adverse effects. According to a case study, an inoperable breast cancer patient received bee venom therapy in addition to traditional chemotherapy. The patient received injections of bee venom. Tumors shrank, and cancer indicators dropped as a result of the therapy.

In patients with advanced sarcomas, another clinical study was performed to evaluate the safety and potential effectiveness of bee venom. Bee venom injections were administered to patients along with chemotherapy, and the results came out to be positive. The bee venom was well tolerated by the patient, and they saw a reduction and stabilization in the cell size of the tumor [4].

Safety considerations while administering bee venom

To reduce and minimize the potential adverse effects of bee venom on patients and ensure their good health, some safety conditions must be ensured before administering bee venom to cancer patients. Allergic reactions are a major concern when talking about a foreign agent as a therapeutic alternative to a disorder. The patient may show a minor local reaction or a serious systemic allergic reaction after the administration of bee venom into their body. It is crucial to check the patient for allergies to bee stings and insect venoms. These patients can be identified with the help of extensive medical history-taking and allergy-testing mechanisms [3].

While administration, the route is fairly considered, as in some cases, bee venom is given subcutaneously, while in others, it is through the intramuscular route. Bee venom acupuncture is also considered in some cases. The chosen route must be in sync with the patient’s present condition as well as his potential allergic reactions to the medication. To minimize the risk of infection, sterile practices must be performed with proper administration techniques.

Bee venom dosage must be determined with caution, keeping in mind the tolerance levels of the patient. To assess the individual’s sensitivity, low doses should be administered first, followed by a gradual increase in the dosage pattern. Close monitoring of the patient should be done to identify any adverse reactions firsthand. Bee venom therapy may be contraindicated in certain situations. For example, individuals with a known history of severe allergic reactions to bee stings or venom should avoid bee venom treatment. Patients with compromised immune systems or certain underlying medical conditions may require cautious evaluation before initiating bee venom therapy. Bee venom therapy should be administered by qualified healthcare professionals trained in its administration and familiar with potential complications. They should have knowledge of appropriate dosage, administration techniques, and emergency management of allergic reactions.

Table 2 shows the summary of the articles used in the review.

**Table 2:** The summary of all articles was used as a reference to write this article.

Author	Year	Findings
Kim W, et al.	2016	The combined effect of morphine and BVA is mediated by 5-HT3 receptors and spinal opioidergic receptors, which provides a strong and long-lasting analgesic effect against neuropathic pain caused by oxaliplatin.
Lee JH, et al.	2001	Management of the edema and discomfort (pain) associated with chronic inflammatory diseases may benefit from bee venom administration.
Oršolić N, et al.	2012	The article states the ability of bee venom and its constituents, such as melittin, to cause apoptosis, immunomodulation, cytotoxicity, and anticancer effects in various tumor cells in vitro or in vivo.
Son DJ, et al.	2007	For various cancers, including prostate and breast cancer, melittin conjugation with hormone receptors and melittin-carrying gene therapy may be helpful as a novel targeted treatment.
Kim W, et al.	2021	The article discusses the medicinal benefits and pharmacological profile of bee venom and its constituent parts.
Apantaku LM, et al.	2000	The article states that when evaluating breast problems, a comprehensive physical examination together with a detailed history of the woman's symptoms and risk factors are crucial. It's also critical to schedule imaging and diagnostic tests appropriately.
Waks AG, et al.	2019	Breast cancer is classified into three main tumor subtypes based on the amplification of the ERBB2 gene and the presence of the estrogen or progesterone receptor. Each of the three subtypes has a unique risk profile and course of treatment.
Kwon N-Y, et al.	2022	This research indicates that certain breast cancer cells are resistant to the anticancer effects of bee venom and its constituent parts. The mechanisms underlying the anticancer effects included cell lysis, cytotoxicity, apoptosis, and gene expression modulation.
Ridner SH, et al.	2013	An overview of the lymphatic system's architecture, physiology, and pathology is given in this article.
Ducic I, et al.	2014	This article states that with breast augmentation, there is a chance of nerve damage, altered feeling, or chronic discomfort. Patients and surgeons might benefit from knowing the prevalence of these disorders. Peripheral nerve surgery may be necessary as part of a multidisciplinary team's prompt treatment plan to maximize patient results.
Lyons JA, et al.	2014	To aid in this decision-making process, we offer a review of the pertinent literature on postmastectomy radiation therapy in this article.
Mignot F, et al.	2022	The study's long-term results appear to be safe; however, locoregional recurrence appears to be slightly higher than reported in the literature, underscoring the need for randomized trials and long-term follow-up for hypofractionated radiation therapy after mastectomy.
Bellik Y, et al.	2015	Current research on the biological and chemical characteristics of the main ingredients in bee venom, as well as the mechanisms of action that underlie these traits, and the reasons why bee venom is seen as a promising alternative treatment.
Choi JH, et al.	2015	The findings of this investigation showed that using bee venom as an anti-MRSA treatment had detrimental effects due to its inherent toxicity. Melittin, the main ingredient in bee venom, on the other hand, shows antibacterial properties with low toxicity both in vitro and in vivo.
Kim S-J, et al.	2012	The findings of this article suggest that apamin is involved in the processing of apoptosis in monocytes and macrophages, which may lead to the development of a medication that prevents atherosclerosis.
Sánchez J, et al.	2017	The article describes the primary bee products' biological characteristics and chemical makeup. The advantageous biological properties of bee products for human health are given particular consideration. Bee venom, bee pollen, royal jelly, honey, and propolis are all utilized in traditional medicine and as food.
Dreyer F, et al.	1990	The article states that the biological characteristics and applications of bee products can be determined by their kind and composition. It will have to reach an understanding regarding the biological role and chemical makeup of the many bee products in relation to issues including pathogen infections, cancer, aging, and age-related illnesses, nutrition, and neurological diseases.
Moga MA, et al.	2018	According to this research, a number of toxins found in snake and bee venom may one day be used to treat ovarian cancer.
Liu C-C, et al.	2016	This research provides an overview of current research on the anticancer effects of bee venom, specifically its primary component, MEL, on several tumor cell types and explain potential anticancer processes.
Zhang S-F, et al.	2017	Because melittin has an anticancer impact on lung cancer cells, it may be used to treat lung cancer both in vivo and in vitro.
Mahmoodzadeh A, et al.	2015	Melittin has a time- and dose-dependent pattern of inhibiting GC-AGS cell growth. Moreover, melittin seems to have induced necrosis in deadly cancer cells.
Tipgomut C, et al.	2018	Melittin's cytotoxicity against ChaGo-K1 cells and its ability to prevent THP-1 cells from differentiating into TAMs make it a viable alternative medication for the treatment of lung cancer.
Giménez D, et al.	2015	The article provides a previously unobserved image of melittin pores generated at lipidic interfaces and provides fresh angles for structural studies of this and other proteins and peptides that form pores employing supported monolayers in the future.
Kim JY, et al.	2015	Apamin inhibits vascular migration and proliferation by blocking the PDGF signaling pathway's G0/G1 cell cycle arrest. Therefore, apamin seems like a good option for treating atherosclerosis.
Modzelewska B, et al.	2003	There are apamin-sensitive K+ channels in non-pregnant women's myometrium. However, based on the results, the possibility cannot be ruled out that non-pregnant women's myometrium contains channels that are sensitive to both cerebrotendinous xanthomatosis and apamin too, comparable to those found in certain smooth muscles.
Cherniack EP, et al.	2018	The two conditions for which bee venom acupuncture shows the most promise are musculoskeletal diseases and Parkinson's disease; nonetheless, well-planned scientific trials are still needed.
Hanson JM, et al.	1974	Vascular endothelium may become anergic to phlogistic stimuli as a direct result of the MCD peptide's anti-inflammatory activity.
Banks BE, et al.	1990	The rat hind paw's inflammatory response was heightened by the injection of both carrageenin and mast cell degranulating peptide.
Trumbeckaite S, et al.	2015	The two main uses of bee products were immune system enhancement and the prevention and treatment of respiratory tract diseases.
Patel S, et al.	2016	The article states the current developments and the extent of modification in cancer treatment, with sufficient focus on the propolis' molecular properties.
Fitzmaurice C, et al.	2017	The article explains that because most cancer prevention initiatives have far wider effects than merely lowering the incidence of cancer, the GBD's capacity to see individual diseases from the standpoint of population health is exceptional and crucial too.

## Conclusions

As described in the article above, breast cancer is among the most dangerous and commonest malignancies in women worldwide. Breast cancer cases and malignancy count are increasing with each passing year due to the changing lifestyle of women nowadays as well as the developments occurring in diagnostic devices consistently. Bee venom as a treatment for breast cancer is an exciting take on the matter that presents exciting possibilities and results. But, due to the limitations of the current evidence, it is essential to approach these findings with caution. The continued efforts of various scientists across the globe will surely provide valuable insights into whether bee venom and its constituents are safe and effective and could potentially become a treatment option for breast cancer in the near future. Overall, in the end, we can say that bee venom might have some hidden potential as a therapeutic agent, but further study is required to understand its clinical utility and its real-life applications.
